# Prevalence and Risk Factors of Low Back Pain in Malaysia: A Scoping Review

**DOI:** 10.21315/mjms2023.30.3.3

**Published:** 2023-06-27

**Authors:** Ab. Hamid Abas, Aziah Daud, Suhaily Mohd Hairon, Mohd Nazri Shafei

**Affiliations:** 1 Department of Community Medicine, School of Medical Sciences, Universiti Sains Malaysia, Kelantan, Malaysia; 2 Training Institute of Ministry of Health Malaysia Sultan Azlan Shah, Perak, Malaysia

**Keywords:** low back pain, Malaysia, scoping review, prevalence, risk factors

## Abstract

Data on the prevalence and risk factors of low back pain (LBP) in Malaysia remain unclear as they are currently limited to specific settings and occupational groups. Therefore, this study aims to determine the prevalence and risk factors of low back pain in Malaysia. In this scoping review, we had systematically searched PubMed, Scopus, ScienceDirect and Google Scholar from January 2016 to April 2020. In addition, we had included cross-sectional studies on LBP in Malaysia. Studies without data on the prevalence and risk factors were excluded. The settings, population, design, sample size, evaluation methods, prevalence and risk factors of the studies were summarised. The literature search identified 435 potentially eligible studies; of these, 21 had met the inclusion criteria. The prevalence of LBP in Malaysia among various types of the population had ranged from 12.4% to 84.6%. Among the various types of occupation, the prevalence of LBP was the highest among nurses (67.9%), followed by drivers (65.7%). In addition, age, gender, body mass index (BMI), lifting heavy objects, working posture, lifestyle, working hours and mental health were identified as the risk factors of LBP in Malaysia. The current evidence suggests that LBP is a serious health problem among several occupational groups in Malaysia. Therefore, it is crucial to implement the correct interventions for the prevention of LBP among these groups.

## Introduction

Low back pain (LBP) is a common musculoskeletal disorder worldwide affecting people of all ages, from children to the elderly. The 2017 Global Burden of Disease Study has estimated that LBP is a leading cause of disability (1). Furthermore, a systematic review published in 2012 showed that the global point prevalence of LBP was 11.9 ± 2.0% and the 1-month prevalence was estimated to be 23.2 ± 2.9% (2). With the aging population, the number of people affected is likely to increase over the coming years.

Several risk factors for LBP that have been identified are age, gender, obesity, smoking, educational level, competitive sports, level of physical activities, duration of electronic device usage, postural habits and psychological factors such as stress, depression and anxiety (3). For example, occupation and lifestyle are the major contributors to the burden of LBP. Occupations with a high physical workload and stress would have greater risk of developing LBP (4, 5). However, the key contributor to the risk factors varies between countries. Therefore, it is important to identify the main contributor of LBP in Malaysia to effectively implement the interventions needed.

Despite the availability of several cross-sectional studies on LBP in Malaysia, the data are currently limited to specific settings and occupational groups (6–8). Given the continuing importance of LBP as a public health problem in various settings and occupational groups, there is a necessity for a scoping review to be done on the available literature to evaluate the prevalence and risk factors of LBP in Malaysia.

## Methods

This study had utilised the established framework of scoping review by Arksey and O’Malley (9). The framework had included five stages: i) identifying the research question; ii) identifying relevant studies; iii) study selection; iv) charting the data and v) collating, summarising and reporting the results (9).

### Identifying the Research Question

The following research questions are addressed in the study:

- What is the prevalence of LBP in Malaysia?
- What are the risk factors of LBP in Malaysia?
- What type of questionnaires are used as the diagnosis methods of LBP in Malaysia?

### Identifying Relevant Studies

We searched the literature for epidemiological studies on LBP in Malaysia between March 2020 and April 2020 according to the guidelines of the preferred reporting items for systematic reviews and meta-analyses that had been modified (10). The research team members used keywords and MeSH terms as search terms to identify the potential studies. Boolean operators (OR, AND NOT), including adjacencies and truncations, were used to combine the keywords and related terms during the literature search. A comprehensive search was performed to identify the primary studies on LBP in Malaysia from January 2016 to April 2020 and the grey literature in which technical reports were included; thus, utilising different electronic databases (PubMed, Scopus, ScienceDirect and Google Scholar).

### Study Selection

The inclusion criteria for the search were published articles dated from January 2016 to April 2020 to ensure the collection of relevant recent data on LBP. Narrative, systematic or other review articles were excluded. The study selection was also limited to Malay and English language articles.

The selection of articles was performed in two stages. In the first stage, researchers had independently screened the titles and abstracts of all the identified resources based on the inclusion criteria and search terms. Then, the researchers had thoroughly screened the selected titles and abstracts to determine the suitability of the content to be included in the review (i.e. met the review’s objectives). Unrelated abstracts were excluded. Finally, the researchers had retrieved the full articles of the selected abstracts.

In the second stage, the full articles were screened to identify items related to the review’s objectives, which would answers the review questions. Similar to the first stage, the researchers had independently reviewed the full articles to determine whether they had met the review’s objectives. To ensure the consistency of the study selection, the data collected by the researchers were compared and the discrepancies between the reviewers were discussed. Data management was done using Mendeley software, version 1.19.2 and the extracted data from the full articles were documented in a Microsoft Excel spreadsheet.

### Charting of Data

Four reviewers had undertaken the final full-text review on the prevalence and risk factors of LBP in Malaysia. Therefore, general and specific information on the studies, such as the author(s), year of publication, study location and settings, study population, study design, sample size, evaluation methods used, prevalence and risk factors, were included in the charting table.

### Collating, Summarising and Reporting the Results

The results of the extracted data were summarised and tabulated. We did not assess the quality of the selected articles, as it was not the objective of this scoping review. Several limitations of the studies were highlighted to address research gaps and provide recommendations for future research on LBP in Malaysia.

## Results

**Figure 1:**
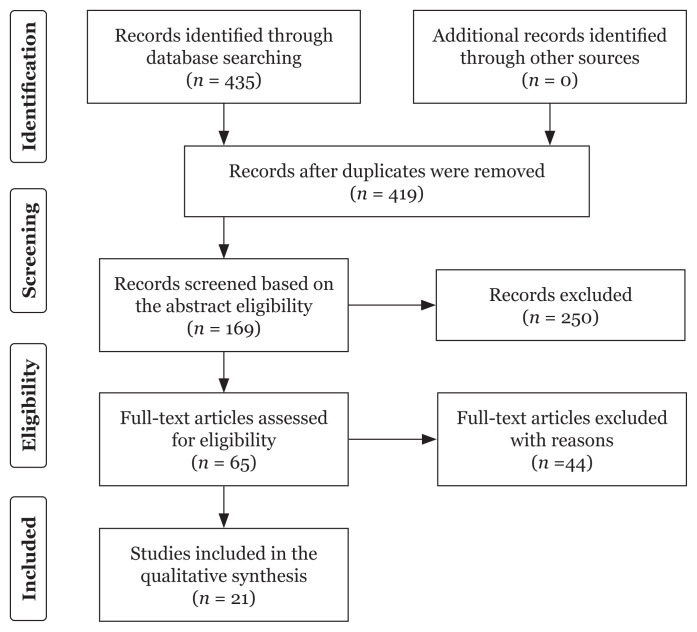
Flowchart of selection process for scoping review of LBP articles

We had retrieved a total of 435 articles from the four databases mentioned. After removing duplicates, we proceeded to screen the titles and abstracts of 419 articles, of which 65 went through for the full-text assessment while the remaining 354 were excluded (Figure 1). In the following full-text assessment, a total of 21 studies on the prevalence and risk factors of LBP in Malaysia had met our inclusion criteria to be included in this review.

### Characteristics of Low Back Pain Literatures in Malaysia

Regarding the geographic distribution of studies, the prevalence of LBP was reported in eight states and two federal territories (Table 1). These include Kelantan, Kuala Lumpur, Selangor, Pulau Pinang, Pahang, Terengganu, Negeri Sembilan, Putrajaya, Sabah and Sarawak. Studies that involved the epidemiology of LBP were mostly conducted in Kelantan with four studies followed by Kuala Lumpur and Pulau Pinang with three studies each. Meanwhile, no studies reported the prevalence of LBP in Johor, Melaka, Kedah, Perak and Perlis.

In the 21 articles reviewed, the studied population was from different types of occupations. Four articles had studied nurses, three articles had included primary and secondary school teachers, two had assessed drivers and the remaining had studied tea plantation workers, elderly care home workers, migrant workers, office workers, shipyard welders, factory workers, doctors, students, army and dental auxiliary. The overall sample size in this study was 6,914 respondents, with a minimum sample of 27 and a maximum of 1,482. Only 11 (50%) of the selected articles stated their study period, in which the majority (10) was conducted between 2012 and 2018. A total of 11 articles did not specify the period of their studies.

### Prevalence of Low Back Pain in Malaysia

The majority (61.9%) of the articles had utilised self-administered questionnaires to evaluate the presence of LBP among the respondents. Four articles had used Nordic musculoskeletal questionnaire (NMQ) while the remaining had utilised: i) the visual analog scale (VAS); ii) a screening tool for neuropathic pain components using a simple patient-based (pain*DETECT*) questionnaire; iii) Cornell musculoskeletal discomfort questionnaire (CMDQ) and iv) a Back Apparatus: a collaborative effort between the Universiti Kebangsaan Malaysia Medical Centre (UKMMC) and Social Security Organisation (SOCSO) (BACKS) tool questionnaire.

The prevalence of LBP in Malaysia among various types of the population had ranged from 12.4% to 84.6%. The highest prevalence is observed among pregnant women in Selangor (11), while the lowest is recorded among patients with LBP at the Universiti Malaya Medical Centre (12). Among the various types of occupation, the prevalence of LBP was the highest among nurses, with an average prevalence of 67.9%, derived from four studies. The second most affected occupation is the drivers, with an average prevalence of 65.7% based on two studies that assessed commercial vehicle drivers in Sabah and ambulance drivers in Kelantan (8, 13).

### Risk Factors of Low Back Pain in Malaysia

Of the 21 included articles, the risk factors were investigated in 18 studies. However, three articles did not determine the risk factors of LBP among their study respondents. Various types of risk factors associated with LBP were reported in these 18 studies. Regarding socio-demographic risk factors, three articles demonstrated a significant association of age, gender and body mass index (BMI) with LBP. The prevalence of LBP shows a significant increase among the 40 years old–49 years old and 50 years old–59 years old groups (14). Furthermore, females have a higher prevalence of LBP than males (7, 15). Those classified as obese based on their BMI also show increased odds of developing LBP (12, 14, 16).

Regarding occupational factors, three articles showed the association between the prevalence of LBP with lifting heavy objects (5, 7, 17). The prevalence of LBP is also significantly associated with working posture, such as prolonged sitting (7, 18) and frequent standing (4, 14, 18). Moreover, personal lifestyle behaviours such as smoking and hobbies are reported as risk factors for LBP (5, 8, 14). Other risk factors of LBP included working hours and mental health (19, 20).

## Discussion

To our knowledge, this study was the first to review the prevalence and risk factors of LBP in Malaysia. Our study showed that active surveillance was conducted in Kelantan, Kuala Lumpur and Pulau Pinang. However, epidemiological data were reported in Johor, Melaka, Kedah, Perak and Perlis. These gaps highlight the need for surveillance in those areas to better understand the epidemiological status of LBP. In addition, the study had demonstrated active surveillance among nurses, teachers and drivers. One of the explanations concerning the interest of Malaysian researchers in studying LBP among these occupational groups may be attributed to the several reports from other countries that highlighted nurses, teachers and drivers as the high-risk group for LBP (21–25).

Various questionnaires had been used to assess the presence of LBP, such as NMQ, VAS, the pain DETECT questionnaire, CMDQ, Malay-validated BACKS tool questionnaire and self-administered questionnaire. These questionnaires would highlight the heterogeneity in the assessment tools used to screen LBP. However, it was difficult to assume the finest instrument out of the available questionnaires as there was a lack of study on the comparison between the evaluation tools. Currently, the gold standard tool to evaluate the presence of LBP still does not exist. Therefore, it is urgently needed so that future studies on LBP in Malaysia can utilise the tool; thus, improving the consistency of the epidemiological data. In addition, it was exceptional to assess the quality of life among the participants suffering from LBP due to the lack of data in this particular field. Therefore, the Short Form 36 Health Survey (SF-36) questionnaire can be applied for this purpose because it consists of all the indicators of overall health status.

The study showed a generally high prevalence of LBP in Malaysia, particularly among nurses and drivers. The findings agreed with the previous study done in other countries such as Bangladesh and South Africa, which also showed a high prevalence of LBP among nurses in private and government hospitals (21, 22). The main reason for the high prevalence of LBP among nurses is the lack of supporting staff, which led to working overtime, frequent standing and manual lifting in the daily job involving patient care (21). In a study carried out in South Africa, it is reported that the high prevalence of LBP among nurses in the district hospital is attributed to the prolonged position, working in a cramped position, bending or twisting and pushing or pulling (22). This is particularly interesting, given the increasing workload among nurses during the coronavirus disease 2019 (COVID-19) pandemic. Due to that, the prevalence of LBP among nurses is likely to increase substantially. According to a systematic review and meta-analysis of 21 randomised clinical trials, it is said that exercise in combination with education is likely to reduce the risk of LBP (26). Therefore, such interventions among nurses are urgently needed to reduce the risk of LBP.

Similarly, a high prevalence of LBP among drivers also have been reported worldwide (27–30). A cross-sectional study which involved 180 public bus drivers carried out in Cairo, Egypt had reported that 73.9% of the public bus drivers experienced LBP (27). In China, a cross-sectional survey involving 800 taxi drivers revealed that 54% had LBP (28). Truck drivers in Modjo Dry Port, Ethiopia are also among the high-risk group for LBP, with a prevalence of 65% out of 400 truck drivers interviewed (29). Studies in China and Egypt identified driving duration as the contributing factor to the high incidence of LBP among the drivers. On the contrary, our study had observed no significant association between driving duration and LBP (13). This finding was similar to a study in Modjo Dry Port, Ethiopia which reported no significant association between driving duration and LBP (29). Inconsistent findings among studies in different geographical areas suggest that the risk factors of LBP among drivers are varied between settings and it is likely due to roads condition and the vehicle used. Interestingly, LBP among drivers in Malaysia is significantly associated with handling heavy loads and smoking (8, 13).

Age, gender and BMI are the most commonly reported risk factors of LBP in Malaysia. Those in the age groups 40 years old–49 years old and 50 years old–59 years old were found to have higher odds of LBP than the younger groups. Three studies in Malaysia had reported a higher prevalence of LBP among females than males (7, 15). The estimation from the Global Burden of Disease Study in 2017 had reported similar findings in which the prevalence of LBP would increase with age and was higher among females than males (31). However, the findings of this study had contradicted the results of a study carried out in Iran, reporting that there is no significant association between LBP and age or gender (32). Meanwhile, a study in Brazil showed that age is a risk factor only for men but not for women (33). Regarding BMI, we had identified three studies in Malaysia that highlighted obesity as a significant risk factor of LBP (12, 14, 16). This significant association between obesity and LBP agrees with the previous studies in the United States and Japan (34, 35). Furthermore, the gain in mass in the upper body area due to obesity would increase the load on the vertebral discs, a plausible reason to develop LBP. These findings suggest that education and exercise guidance to avoid obesity must be considered for the prevention and treatment of LBP.

This study has several limitations. First, the sample size to estimate the prevalence of LBP was too small in some studies. The smaller sample size might cause inaccurate estimation of the correct prevalence, increasing the margin of errors. Future surveillance on LBP should consider the correct calculation of the minimum sample size so that the reported prevalence would represent the study population. Second, almost 50% of the included studies did not mention the surveillance period. Due to this, we could not accurately estimate the trend of LBP in Malaysia. Finally, the majority of the studies had used self-administered questionnaires to collect the data. This might lead to biasness as the validity and reliability of the questionnaires were not reported. Therefore, we would suggest future surveillance to utilise the commonly used questionnaires such as the NMQ to assess LBP or report the validity and reliability of the self-administered questionnaires.

## Conclusion

LBP is a common problem among nurses, teachers and drivers in Malaysia. With the aging populations, the prevalence of LBP is likely to increase substantially over the coming years. Further research is needed to determine the prevalence and risk factors of LBP in Johor, Melaka, Kedah, Perak and Perlis to better understand the epidemiological status. The available evidence suggested that age, gender and BMI were the significant risk factors of LBP in Malaysia. Meanwhile, lifting heavy objects, prolonged sitting and frequent standing were the most common occupation-related risk factors of LBP in Malaysia. Interventions to prevent LBP among the high-risk groups in Malaysia are urgently needed to improve individuals’ quality of life and increase productivity.

## Figures and Tables

**Table 1:** Characteristics of the prevalence and risk factors of low back pain articles in Malaysia included in the scoping review (*n* = 21).

Authors	Year	Study design	Study setting	Study population	Sample size	Diagnosis methods	Prevalence	Risk factors
(36)	2006	Cross-sectional	Pahang	Tea plantation workers	106	Self-administered questionnaires	64.20%	Feeling the need to work as fast as possible
(15)	2016	Cross-sectional	West coast Malaysia	Elderly care home workers	252	Self-administered questionnaires	33.80%	Female and the burden of manual task
(7)	2015	Cross-sectional	Putrajaya	Secondary school teachers	120	Nordic musculoskeletal questionnaire	72.90%	Prolonged sitting, walking up and down the stairs and lifting loads with hands
(37)	–	Cross-sectional	Sarawak	Nurses working at medical and surgical discipline at a general hospital in Sarawak	141	Self-administered questionnaires	63.10%	Age, years of working experience and history of falls
(4)	–	Cross-sectional	Kelantan	Nurses in critical care units in Hospital Universiti Sains Malaysia	110	Self-administered questionnaires	68.20%	Working experience in the current ward, nursing experience, age and frequent standing
(11)	2012	Cross-sectional	Selangor	Pregnant women who attended the antenatal clinic in Universiti Kebangsaan Malaysia Medical Centre	358	Visual analogue scale (VAS)	84.60%	Type of occupation and history of back pain
(12)	–	Cross-sectional	Kuala Lumpur	Low back patients at the Universiti Malaya Medical Centre	210	The pain DETECT questionnaire	12.40%	Non-Chinese ethnicity, higher body mass index and pain radiation below the knee
(8)	–	Cross-sectional	Kelantan	Ambulance workers in the emergency department in all government hospitals in Kelantan	168	Self-administered questionnaires	65.00%	Gender, smoking status and hobbies
(20)	2014	Cross-sectional	Penang	Secondary school teachers	1482	Self-administered questionnaires	48.00%	Depression, anxiety, high psychological job demand, low skill discretion and poor mental health
(38)	2014	Cross-sectional	Kelantan	Nurses	60	Self-administered questionnaires	63.80%	Race, number of patients to handle, satisfaction with the working environment and social problems
(39)		Cross-sectional	Kuala Lumpur	Filipino migrant workers in Malaysia	60	Nordic musculoskeletal questionnaire	60.00%	–
(16)	2015	Cross-sectional	Kuala Lumpur	Office workers	752	Cornell musculoskeletal discomfort questionnaire (CMDQ)	60.60%	BMI
(40)	–	Cross-sectional	Malaysia	Doctors	86	Self-administered questionnaires	19.80%	–
(17)	–	Cross-sectional	Malaysia	Shipyard welders	27	Modified Nordic musculoskeletal questionnaire	70.40%	Working posture, repetitive work and lifting of heavy objects during welding
(5)	2018	Cross-sectional	Selangor	Malaysian army personnel deployed in Klang Valley	321	Self-administered questionnaires	48.29%	Smoking status, history of LBP, history of accident, military rank, category of the regiment, lifting weights, pushing weights, pulling weights and job-related physical activity
(6)	2016	Cross-sectional	Public hospitals of Pulau Pinang	Nurses	1292	Malay-validated BACKS tool questionnaire	76.50%	Working more than seven hours, twisting of the body while working, manual handling of patients in wards and fatigue
(13)	–	Cross-sectional	Sabah	Commercial vehicle drivers	110	Self-administered questionnaire and the VI-400Pro human vibration monitor	66.40%	Frequent manual handling of heavy loads and awkward posture during manual materials handling
(19)	–	Cross-sectional	Pulau Pinang	Factory workers	626	Self-administered questionnaires	18.70%	Working hours
(14)	–	Cross-sectional	Terengganu	Female teachers in primary school	212	Self-administered questionnaires	25.00%	Age, BMI, sports activity, shoe type, teaching hours and standing hours during school
(18)	2018	Cross-sectional	Negeri Sembilan	Undergraduate medical students of thr International Medical University	339	Self-administered questionnaires	25.40%	Body posture, bad mattress, sitting for long hours and standing at university and carrying backpacks
(41)	2015	Cross-sectional	Kelantan	Dental auxiliary at Hospital Universiti Sains Malaysia	82	Nordic musculoskeletal questionnaire	47.00%	–

Note: (–) = Information not available
